# Finite Element Analysis of Extraocular Muscle and Adipose-Dominant Pathogenesis in Thyroid Eye Disease With Clinical Implications

**DOI:** 10.1167/tvst.15.9.22

**Published:** 2026-09-30

**Authors:** Zexi Sang, Pei Wang, Ru Lin, Xiuhong Li, Li Zhu, Yan Xun, Hongfeng Yuan

**Affiliations:** 1 Aier Academy of Ophthalmology, Central South University, Changsha, China; 2 Department of Ophthalmology, Chongqing Aier Eye Hospital, Chongqing, China; 3 Aier Eye Medical Center, Anhui Medical University, Hefei, China

**Keywords:** Graves’ ophthalmopathy, finite element analysis, dysthyroid optic neuropathy, biomechanics, orbital diseases

## Abstract

**Purpose:**

To compare the biomechanical mechanisms of muscle-predominant and adipose-predominant thyroid eye disease (TED) using a three-dimensional (3D) finite element analysis (FEA) model and validate findings through clinical outcomes.

**Methods:**

A 3D orbital FEA model was reconstructed from healthy computed tomography imaging. Extraocular muscle hypertrophy and adipose proliferation were simulated under fixed volumetric expansion. Optic nerve (ON) stress and globe displacement were measured across six simulation groups. For clinical validation, 36 patients with TED were retrospectively categorized as adipose type (AT) or muscle type (MT) based on muscle-to-orbital area ratios to compare dysthyroid optic neuropathy (DON) incidence.

**Results:**

Medial rectus hypertrophy generated the highest single-muscle ON stress (0.59 kPa). Muscle-predominant simulations produced higher peak ON stress than adipose-predominant simulations (1.40 vs. 0.18 kPa). Conversely, adipose expansion caused much greater globe displacement (4.34 vs. 1.53 mm), reflecting adipose-driven exophthalmos. Clinically, MT patients demonstrated a significantly higher DON rate than AT patients (83.3% vs. 22.2%, *P* < 0.001), aligning with FEA-predicted risk patterns.

**Conclusions:**

Muscle-predominant TED is the primary biomechanical driver of apical compression and DON risk. Adipose-predominant TED primarily causes exophthalmos with lower tractional stress on the optic nerve. Medial rectus hypertrophy is the most significant individual contributor to compressive load.

**Translational Relevance:**

These biomechanical phenotypes help clinicians identify high-risk patients with DON (MT) who require urgent apical decompression, whereas AT patients may benefit more from volume reduction strategies focused on proptosis correction.

## Introduction

Thyroid eye disease (TED), also known as Graves’ orbitopathy, is the most common autoimmune disorder involving the adult orbit and represents the principal extrathyroidal manifestation of Graves’ disease.^1^ Its pathophysiology is characterized by an autoimmune response that leads to inflammatory infiltration, glycosaminoglycan accumulation, and progressive enlargement of orbital soft tissues, particularly the extraocular muscles (EOMs) and the intraorbital adipose tissue.^2^ Because the orbit is a rigid bony cavity with limited capacity for expansion, such tissue enlargement causes a rapid rise in intraorbital pressure. This pressure increase results in a wide range of clinical manifestations, including proptosis, eyelid retraction, restrictive strabismus, and diplopia.^3^ The most vision-threatening complications are exposure keratopathy and dysthyroid optic neuropathy (DON), both of which may lead to permanent visual loss.^4^ Orbital decompression is commonly performed in cases of DON, severe corneal exposure, or cosmetically significant proptosis caused by elevated orbital pressure.^5^^,^^6^

Marked heterogeneity in the pathologic presentation of TED is frequently noted in clinical practice. Some patients show predominant expansion of intraorbital fat with relatively mild involvement of the extraocular muscles, whereas others demonstrate clear muscular hypertrophy often accompanied by motility restriction.^7^ To account for this variability, a two-type clinical classification has been proposed, distinguishing a fat-predominant form without significant motility impairment from a muscle-predominant form characterized by enlarged EOMs and functional limitation.^8^ Although not universally adopted, this classification highlights an important observation: the degree of extraocular muscle involvement is closely associated with disease severity, prognosis, and the risk of DON. Multiple clinical studies have shown that DON occurs more frequently in patients with marked EOM hypertrophy than in those whose disease is dominated by adipose expansion.^2^^,^^3^ Despite this consensus, the biomechanical basis of this difference has not been clearly or quantitatively clarified.^9^ Key questions remain unresolved, including why muscular hypertrophy produces a greater compressive threat to the optic nerve than adipose expansion under the same relative volumetric enlargement and how these two patterns differ in their ability to induce proptosis or transmit stress to the optic nerve. The lack of answers to these fundamental biomechanical questions limits accurate risk stratification and restricts the development of individualized surgical strategies such as choosing between fat decompression and bony decompression.

Addressing these gaps requires analytical tools capable of simulating and quantifying the mechanical environment within complex biological structures. Finite element analysis (FEA) is a mature computational biomechanics method that provides an effective solution.^10^ By reconstructing patient-specific three-dimensional (3D) orbital models from computed tomography (CT) images, FEA enables numerical calculation of stress, strain, and displacement distributions under defined loading conditions.^11^ Compared with cadaveric studies, FEA offers several advantages: it is noninvasive, reproducible, cost-effective, and suitable for controlled parameter variation, which allows detailed investigation of biomechanical phenomena that cannot be accessed through clinical observation or in vitro experiments.^12^ Recent studies have further demonstrated its translational potential. Wu et al.^13^ showed that augmented endoscopic decompression achieves greater reduction in intraorbital pressure than conventional techniques. Jeong et al.^14^ emphasized the value of patient-specific FEA in planning surgery for muscle-predominant disease. Jafari et al.^15^ developed a constitutive model of the extraocular muscles and quantified the mechanical load transmitted to the optic nerve during muscle contraction.

On the basis of these advances, the present study aims to construct a 3D finite element model of the orbit to quantitatively compare the pathogenic effects of EOM hypertrophy and intraorbital adipose proliferation. The model evaluates their respective influences on globe displacement and stress distribution within the intraorbital segment of the optic nerve and seeks to identify the biomechanical pattern most strongly associated with the development of DON. The simulation results are then correlated with clinical data to ensure translational relevance. Ultimately, this study aims to provide objective and quantitative evidence to support individualized diagnosis and treatment planning for patients with TED.

## Materials and Methods

### Study Participants and Data Collection

This study was conducted in accordance with the principles of the Declaration of Helsinki. The finite element model was constructed using anonymized medical imaging data from a healthy adult volunteer. The retrospective study protocol used for clinical validation was approved by the Ethics Committee of Chongqing Aier Eye Hospital (Approval No. 202510030), and informed consent was waived.

A high-resolution CT scan of the head and neck from a healthy 28-year-old female volunteer (slice thickness 0.625 mm) was used to construct the geometric model. The 0.625-mm slice thickness provided sufficient resolution for reconstructing the major orbital structures and rectus muscle contours used in this study. Segmentation was performed using multiplanar review and manual correction to reduce partial-volume error. Nevertheless, resolution may still limit accuracy of small structures and superior–inferior boundary definition, which was considered when interpreting the model. For clinical validation, complete clinical records and orbital CT data were retrospectively collected from 36 patients diagnosed with TED at our institution between December 2022 and December 2024. The inclusion criteria were (1) a clinical diagnosis of TED, (2) complete and diagnostically adequate orbital CT imaging, and (3) documentation of extraocular motility and diplopia assessment. The exclusion criteria were (1) a history of orbital surgery; (2) comorbidities affecting orbital anatomy such as tumors, trauma, or congenital abnormalities; and (3) significant CT artifacts precluding accurate measurement. All included patients underwent orbital CT scans.

To explore the correlation between clinical heterogeneity of TED (adipose-predominant and muscle-predominant involvement) and the risk of DON, the 36 patients with TED were grouped using a combination of imaging and clinical functional assessments.

DON was diagnosed by an experienced orbital specialist based on thyroid-related optic nerve dysfunction that could not be explained by other ocular diseases. Diagnostic evidence included decreased best-corrected visual acuity, impaired color vision, visual field defects, relative afferent pupillary defect, optic disc edema or pallor, and compatible radiologic findings such as orbital apical crowding or optic nerve compression. Patients were classified as having DON when clinical evidence of optic nerve dysfunction was present together with supportive orbital imaging findings.

The degree of extraocular muscle hypertrophy was quantified using the ratio of extraocular muscle area (EMA) to orbital area (OA) described by Kuriyan et al.^16^ To ensure consistency, all measurements were performed on a standardized coronal CT plane. This plane was identified using multiplanar reconstruction to ensure perpendicular alignment with the optic nerve axis, and a slice located near the posterior pole of the globe was selected. Using the RadiAnt DICOM Viewer (Medixant, Poznań, Poland), the orbital bony contour was manually traced to obtain the OA, and all visible extraocular muscles were outlined to obtain the total EMA (Figs. 1A, 1B). The muscles included the superior rectus, inferior rectus, medial rectus, lateral rectus, and the levator complex. Since the superior rectus and levator muscles cannot be reliably separated on this plane, they were measured as a single anatomic unit. EMA was calculated as the sum of the cross-sectional areas of all outlined muscles. All EMA and OA measurements were performed twice by the same trained investigator using the same standardized coronal CT plane and tracing protocol. The mean of the two measurements was used for subsequent calculation of the EMA/OA ratio and statistical analysis.

**Figure 1.**
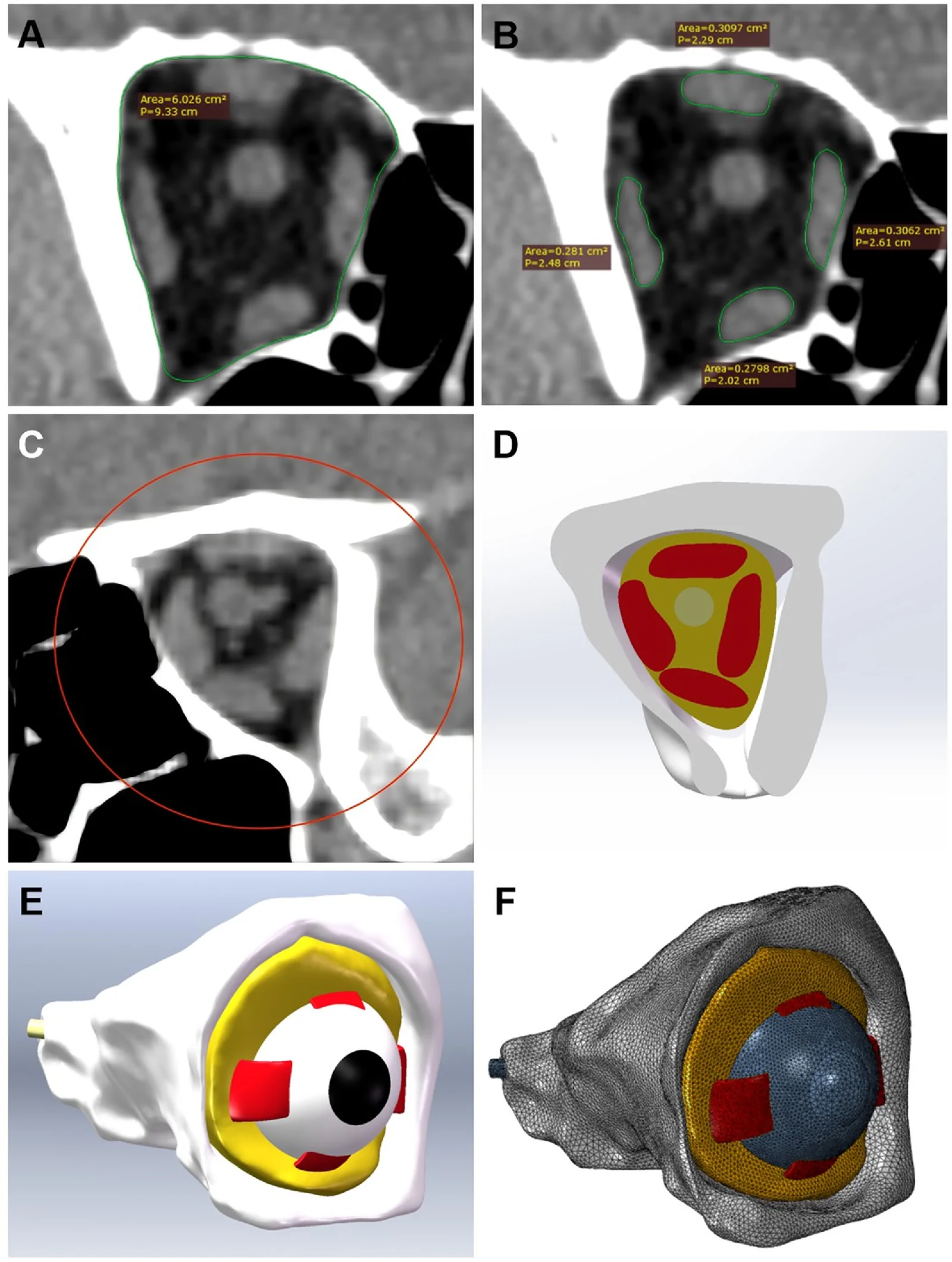
Quantitative imaging analysis and 3D finite element model construction process. (**A**) In the standardized coronal CT image, the orbital contour (*green line*) is manually traced along the inner margin of the orbital bone to measure the OA. (**B**) On the same plane, the cross sections of the rectus muscles (*green line*) are manually outlined, and their areas are summed to calculate the EMA. (**C**) Original high-resolution orbital CT scan (coronal view) used for geometric reconstruction, with the *red circle* indicating the anatomic structure of the orbital apex. (**D**) Reconstructed 3D geometric model (coronal section at the orbital apex), including the orbital bone (*white*), intraorbital adipose tissue (*yellow*), four rectus muscles (*red*), and optic nerve (*central*). (**E**) Complete assembled geometric model with the globe (front-lateral view). (**F**) Final finite element mesh model, showing the mesh division of all components.

To establish classification thresholds, orbital CT scans from 10 adult controls were additionally collected. The inclusion criteria were (1) CT performed for nonorbital indications with radiologically normal orbits and (2) no history of thyroid disease, orbital disease, trauma, or surgery. The exclusion criteria were (1) any orbital structural abnormalities on CT and (2) clinical suspicion of systemic diseases affecting the orbit. EMA and OA were measured using the same protocol, and EMA/OA values were used to calculate the mean and standard deviation for the control group. The normal range was defined as the mean ± two standard deviations. Functional assessment focused on the presence of diplopia caused by restrictive strabismus. In accordance with Nunery's classification, functional diplopia is defined as constant diplopia occurring within the primary gaze and up to 20° of ocular excursion.^17^

Based on combined imaging and functional assessments, the 36 patients with TED were categorized into an adipose-predominant type (hereinafter referred to as AT) and a muscle-predominant type (hereinafter referred to as MT). AT patients were required to meet both criteria: absence of functional diplopia in primary gaze and within 20° of gaze excursion and an EMA/OA value within the normal range defined by the control group. MT was defined by either the presence of functional diplopia or an EMA/OA value exceeding the control mean by more than two standard deviations. Complete medical records were collected for all patients, with specific documentation of DON for subsequent correlation analyses.

### Overall Study Design

This study consisted of a finite element simulation component and a clinical validation component. First, a high-resolution orbital CT scan from a healthy adult volunteer was used to reconstruct a standardized 3D orbital finite element model. Second, orbital CT scans from 10 radiologically normal adult controls were used to establish the reference range of the EMA/OA ratio. Third, 36 patients with TED were retrospectively classified into adipose-predominant and muscle-predominant subtypes using the EMA/OA ratio and functional motility criteria, and DON incidence was compared between the two subtypes.

### Construction of the 3D Finite Element Model

Orbital CT data were imported in DICOM format into medical image processing software (Mimics 21.0; Materialise, Leuven, Belgium). Segmentation of orbital structures was performed using predefined grayscale thresholds, allowing semiautomatic reconstruction of the 3D orbital anatomy (Fig. 1C). The bony orbit and intraorbital adipose were exported as geometric models, which were then smoothed, denoised, and converted into surface meshes before being imported into SolidWorks (Dassault Systèmes, Vèlizy-Villacoublay, France).

To standardize and control the morphology of key anatomic components, direct measurements were obtained from the CT images, including globe dimensions, optic nerve length and cross-sectional size, and the length, orientation, and cross-sectional parameters of the four rectus muscles. These measurements were used to parametrically reconstruct the globe, optic nerve, and rectus muscles in SolidWorks (Figs. 1D, 1E). The CT-derived surface meshes of the bony orbit and intraorbital adipose tissue were then integrated with these parametrically reconstructed structures to form a hybrid geometric model. In this model, the CT-derived meshes defined the anatomic boundaries of the orbital cavity and intraorbital soft tissue space, whereas parametric reconstruction improved the geometric regularity and mesh quality of the globe, optic nerve, and rectus muscles and facilitated controlled tissue expansion. Although direct use of CT-derived meshes for all structures could better preserve individual anatomic details, it would introduce additional geometric irregularity and interstructural variability, thereby reducing the ability to isolate the independent biomechanical effects of muscle and adipose enlargement under standardized conditions.

The superior and inferior oblique muscles were not reconstructed as independent model components because their complex trajectories and irregular morphology pose challenges for high-fidelity reconstruction.^18^ No separate material properties or expansion loads were assigned to these muscles; instead, their anatomic regions were treated as part of the surrounding intraorbital adipose tissue compartment. Consequently, their independent mechanical contribution was not simulated. Their hypertrophy is also less prominent in TED, and this study focused on the compressive effects exerted by the rectus muscle cone at the orbital apex.^19^

All reconstructed components, including the bony orbit, adipose, globe, optic nerve, and rectus muscles, were assembled in SolidWorks to form the complete geometric model for finite element analysis. The model was then imported into HyperMesh (Altair, Troy, MI, USA) for high-quality meshing. To enhance computational accuracy and prevent shear locking during large deformations, modified quadratic elements were applied to all orbital soft tissues, including the globe, optic nerve, orbital adipose, and EOMs. The orbital bone was modeled using linear tetrahedral elements to optimize computational efficiency without compromising the mechanical response of the inner soft tissues, and mesh convergence testing was performed. The final model consisted of 886,374 elements and 815,929 nodes (Fig. 1F). After meshing, the complete model was exported to Abaqus 2020 (Dassault Systèmes) for numerical simulation. The model was designed as a controlled mechanistic model to compare relative biomechanical effects under standardized conditions, rather than as a patient-specific predictor of absolute stress values.

### Material Properties and Biomechanical Simulation

In the biomechanical definition, the bony orbit was treated as a rigid structure. In the finite element model, the bony orbit was fixed in all degrees of freedom and served as the rigid boundary of the orbital cavity. The globe, optic nerve, rectus muscles, and intraorbital adipose tissue were assembled within the orbital boundary according to their reconstructed anatomic relationships. Contact or constraint relationships among adjacent soft tissues were defined to allow mechanical load transmission during tissue expansion while preventing nonphysiological penetration. This assumption is justified by the large difference in stiffness between orbital bone, with a Young's modulus of approximately 1.45 × 10⁴ MPa, and the soft tissues, all of which have values below 1 MPa. Treating the bone as rigid reduces computational cost without affecting the mechanical response of the soft tissues. All degrees of freedom of the orbital bone were fixed to represent its physiological constraint.

To accurately capture the nonlinear stress–strain behavior of orbital tissues under significant enlargement, the EOMs and intraorbital adipose were modeled as hyperelastic materials. Specifically, the mechanical response of these tissues was characterized by a strain energy potential (*W*).

For the intraorbital adipose, a neo-Hookean model was implemented to define its isotropic hyperelasticity:

$$\begin{array}{c} W=C_{10}\left({\bar{I}}_{1}-3\right)+\frac{1}{D_{1}}{\left(J-1\right)}^{2} \end{array}$$

where *C*_10_ is a material constant equal to one-half of the initial shear modulus, $\bar{I}{}_{1}$ is the first deviatoric strain invariant, *D*_1_ is the compressibility parameter, and *J* is the elastic volume ratio.

For the EOMs, a first-order Ogden model was utilized to capture the tissue's specific nonlinear behavior:

$$\begin{array}{c} W=\frac{2\mu }{\alpha^{2}}\left({\bar{\lambda }}_{1}^{\alpha }+{\bar{\lambda }}_{2}^{\alpha }+{\bar{\lambda }}_{3}^{\alpha }-3\right)+\frac{1}{D}{\left(J-1\right)}^{2} \end{array}$$

where *µ* and *α* are the temperature-dependent material constants, ${\bar{\lambda }}_{1}^{\alpha }$ are the deviatoric principal stretches, and *D* is the compressibility coefficient.

The specific material constants used in these simulations were derived from validated biomechanical studies of human soft tissues to ensure physiological relevance within the Abaqus framework.

Material parameters, including Young's modulus and Poisson's ratio, were assigned according to published literature^20^^–^^22^ (Table 1).

**Table 1.** Material Properties

Material	Constitutive Model	Material Parameters	Density (g/cm^3^)	Expansion Coefficient		
Orbital bone	Linear elastic	*E =* 1.45 × 10^4^, *v* = 0.35	1.60	—		
Eyeball	Linear elastic	*E =* 0.50, *v* = 0.40	1.07	—		
Optic nerve	Linear elastic	*E =* 0.50, *v* = 0.47	1.01	—		
Intraorbital adipose	Neo-Hookean	*C*_10_ *=* 5.1 × 10^−6^, *D_1_* = 100	0.98	0.17		
Extraocular muscle	Ogden (*n* = 1)	*µ* = 3.43 × 10^−3^, *α* = 8.74	1.05	0.47 (α_11_)	0.014 (α_22_)	0.024 (α_33_)

*E*, *C*_10_, and μ are expressed in MPa; *D*_1_ is expressed in MPa^−1^; Poisson's ratio and the Ogden exponent alpha are dimensionless.

Tissue enlargement in TED was simulated using an expansion method. A virtual temperature field was applied in designated analysis steps, with an assigned expansion coefficient producing controlled volumetric expansion.^23^ Orbital adipose was modeled with isotropic expansion, while muscle hypertrophy was modeled with anisotropic expansion aligned with the fiber direction to more accurately reflect pathologic changes.^24^ For muscle hypertrophy, anisotropic expansion was applied along the principal fiber direction of the target rectus muscle using predefined expansion coefficients. In the single-muscle hypertrophy groups, expansion was applied only to the designated rectus muscle, whereas no expansion load was applied to the remaining rectus muscles. In the combined rectus hypertrophy group (CRG), the same expansion protocol was applied simultaneously to all four rectus muscles. In the adipose hyperplasia group (AG), isotropic expansion was applied to the entire intraorbital adipose compartment.

For consistency, the final-to-initial volume ratio was fixed at 1.67 in all simulation groups (Fig. 2). This standardized target volume ratio enabled comparison of the biomechanical responses to different tissue enlargement patterns under the same relative volumetric increase. The value of 1.67 was selected as a controlled comparative loading condition and was not intended to represent a universal biological growth ratio, a clinically defined threshold, or a patient-specific degree of tissue enlargement.

**Figure 2.**
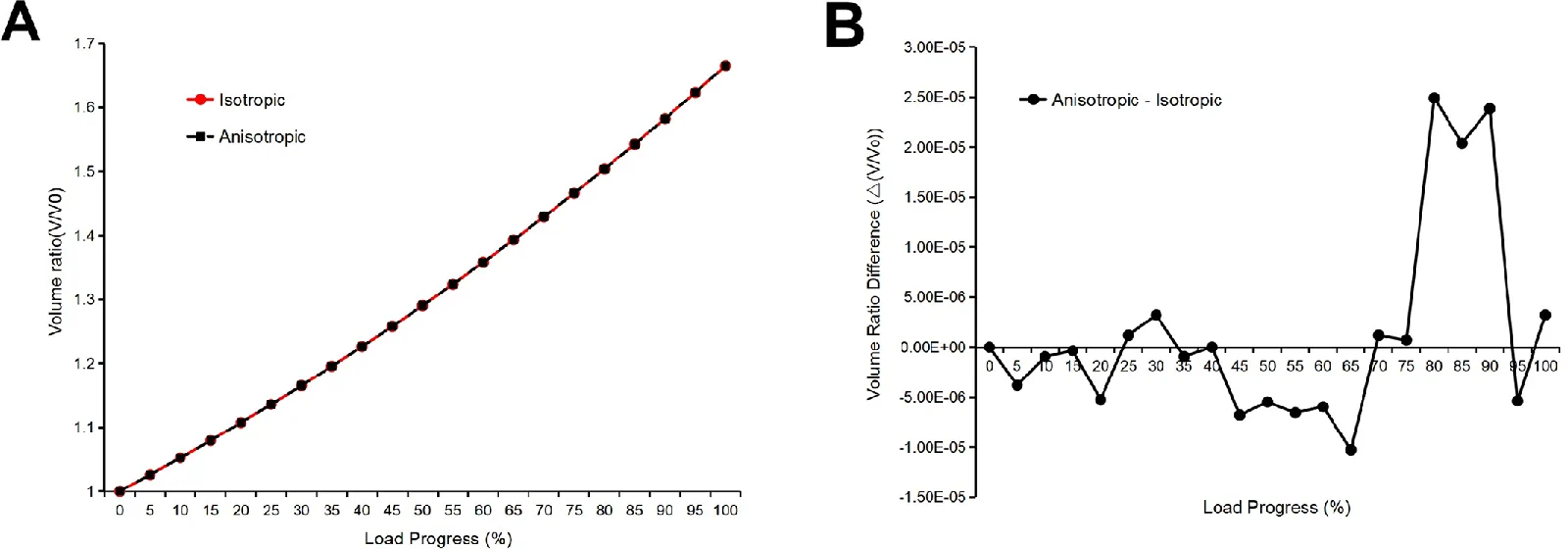
Validation of the effectiveness of expansion simulation method. (**A**) The change in the volume ratio with load progress under both isotropic and anisotropic expansion settings, showing that the two curves highly overlap. (**B**) The volume ratio difference between the two simulation methods throughout the loading process, demonstrating that their numerical differences remain within an insignificant range.

### Simulation Design and Evaluation Metrics

To compare the pathogenic effects of different patterns of tissue enlargement, six simulation groups were constructed: a medial rectus hypertrophy group (MRG), an inferior rectus hypertrophy group (IRG), a superior rectus hypertrophy group (SRG), a lateral rectus hypertrophy group (LRG), CRG, and AG. A mixed pattern combining both muscle and adipose enlargement was not included to isolate and compare the independent biomechanical effects of each proliferative mechanism.

The simulation design allowed quantitative evaluation at two levels. Comparison between CRG and AG provided a direct assessment of biomechanical differences between the two major clinical patterns in terms of optic nerve compression and proptosis, thereby identifying the high-risk biomechanical profile associated with DON. The four individual rectus groups (MRG, IRG, SRG, LRG) were used to determine which muscle contributes most strongly to optic nerve compression in muscle-predominant disease, providing a quantitative explanation for known clinical observations.

Two core biomechanical metrics were extracted. To evaluate the risk and mechanisms of optic nerve compression, the maximum von Mises stress and the corresponding stress distribution patterns on the optic nerve were measured. In addition, sagittal displacement of the corneal apex was recorded to quantify the efficiency of different enlargement patterns in producing proptosis.

## Results

### Independent Rectus Muscle Hypertrophy

To explore the compressive mechanisms of different tissue proliferations, this study first analyzed the biomechanical effects of the MRG, SRG, LRG, and IRG groups under the same volume expansion ratio. As shown in Figure 3, all independent rectus muscle groups caused direct mechanical compression on the optic nerve at the orbital apex. The stress cloud maps, including the coronal section at the orbital apex and the lateral view of the optic nerve, clearly show that the locations of the high-stress regions are closely related to the anatomic positions of the proliferated muscles: MRG (Fig. 3A) and LRG (Fig. 3C) produce high-stress zones on the medial and lateral sides (i.e., horizontal direction) of the optic nerve, respectively, while SRG (Fig. 3B) and IRG (Fig. 3D) create high-stress regions above and below the optic nerve (i.e., vertical direction). Quantitative data from the peak stress values (Fig. 4C) also demonstrate this anatomic correlation. Among the four independent rectus muscles, MRG generates the highest stress value (0.59 kPa), followed by SRG (0.51 kPa), LRG (0.44 kPa), and IRG (0.34 kPa). The peak stress in MRG is higher than the lowest stress observed in IRG.

**Figure 3.**
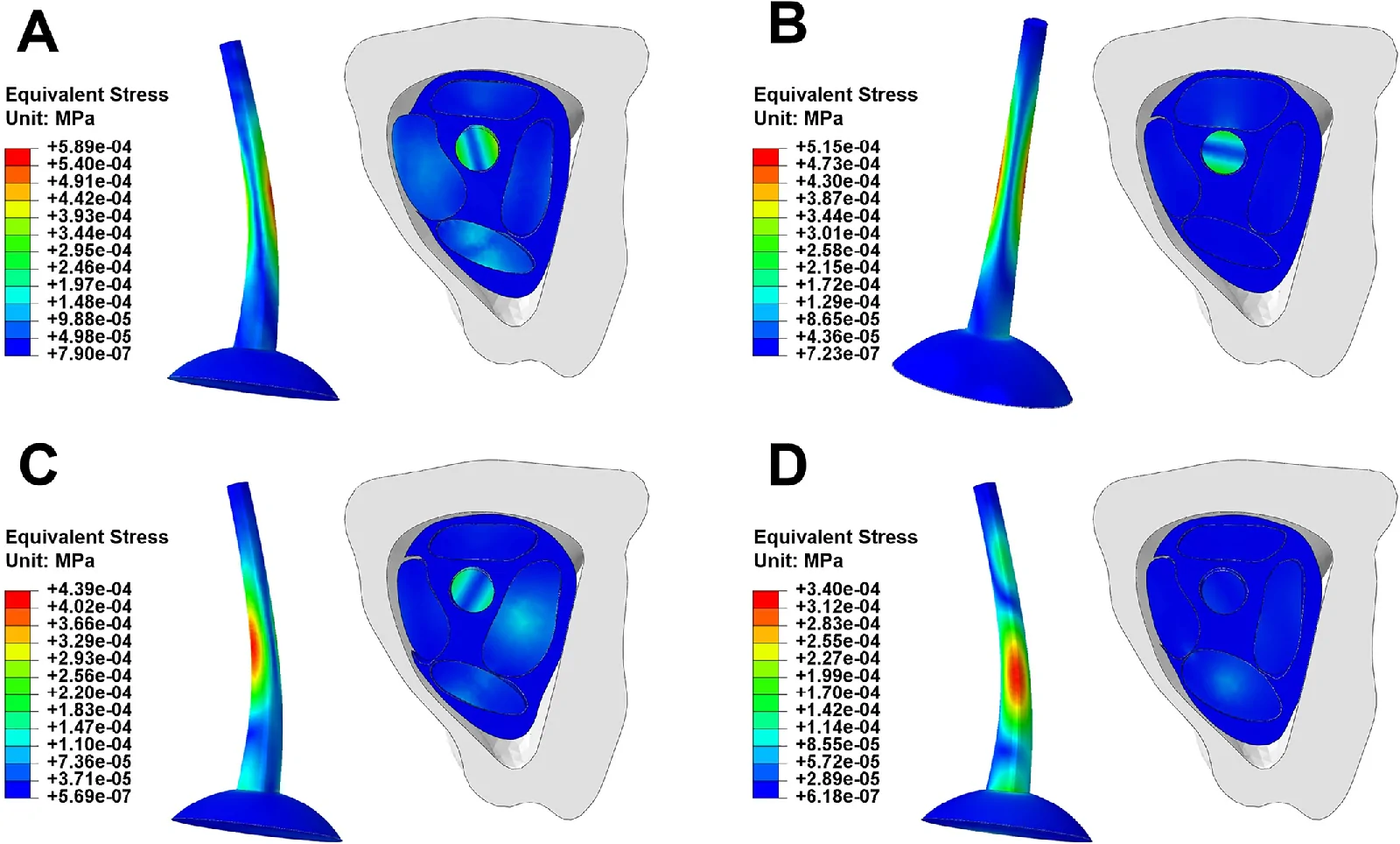
Finite element stress analysis of independent rectus muscle hypertrophy. All stress cloud maps are derived under the same volume expansion ratio and include the coronal section at the orbital apex (*right*) and the anterior-lateral view of the optic nerve (*left*). (**A**) MRG von Mises stress distribution map, showing that the high-stress region is concentrated in the horizontal direction of the optic nerve. (**B**) SRG von Mises stress distribution map, showing that the high-stress region is concentrated in the vertical direction of the optic nerve. (**C**) LRG von Mises stress distribution map, showing that the high-stress region is concentrated in the horizontal direction of the optic nerve. (**D**) IRG von Mises stress distribution map, showing that the high-stress region is concentrated in the vertical direction of the optic nerve.

**Figure 4.**
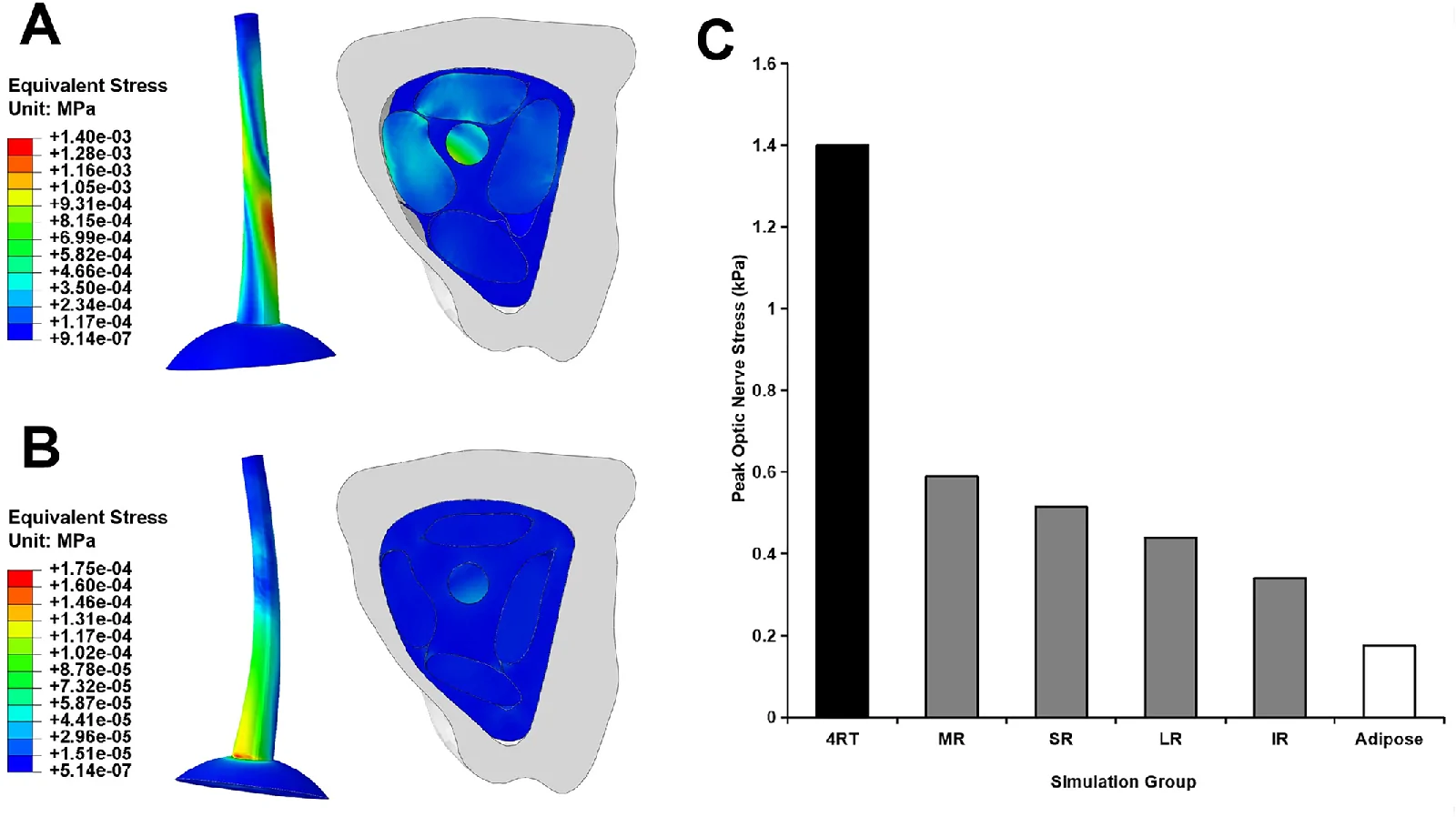
Stress analysis and quantification summary for CRG and AG. The stress cloud maps (**A**, **B**) include the coronal section at the orbital apex (*right*) and the anterior-lateral view of the optic nerve (*left*). (A) CRG von Mises stress distribution map, showing high stress concentrated at the orbital apex, indicating direct compression by the muscle cone. (**B**) AG von Mises stress distribution map, showing high stress concentrated at the optic nerve–globe connection (optic nerve head), indicating a pulling effect. (**C**) Bar chart summarizing the peak optic nerve stress (kPa) for all six simulation groups. In panel **C**, 4RT denotes CRG; MR, SR, LR, and IR denote MRG, SRG, LRG, and IRG, respectively; and Adipose denotes AG.

### Biomechanical Effects of the Two Major Subtypes

Additionally, this study compared the simulation results of two different pathogenic types, CRG and AG. Stress analysis (Fig. 4) revealed differences in the compressive mechanisms between the two patterns. The stress distribution of CRG (Fig. 4A) is similar to the independent rectus muscle groups, showing concentrated compression at the orbital apex on the optic nerve by the muscle cone. In contrast, AG (Fig. 4B) exhibits a stress concentration at the optic nerve–globe connection (i.e., the optic nerve head), suggesting that the main mechanism is the forward displacement of the globe due to intraorbital adipose tissue expansion, which generates a pulling effect on the optic nerve. The bar chart of peak stress (Fig. 4C) clearly shows that CRG produces a peak stress (1.40 kPa) much higher than AG (0.18 kPa).

Displacement analysis (Fig. 5) shows differences in the degree of proptosis between the two subtypes. The displacement cloud maps visually indicate that at the end of the simulation loading, the globe displacement caused by AG (Fig. 5A) is much larger (4.34 mm) than in CRG (Fig. 5B) (1.53 mm). The displacement curves over the entire loading process (Fig. 5C) also show that the globe displacement in AG accelerates nonlinearly with increasing tissue growth, while CRG's increase remains relatively gradual. Based on the final simulation data, AG (adipose proliferation) exerts about 2.84 times the effect on globe displacement compared to CRG (muscle hypertrophy).

**Figure 5.**
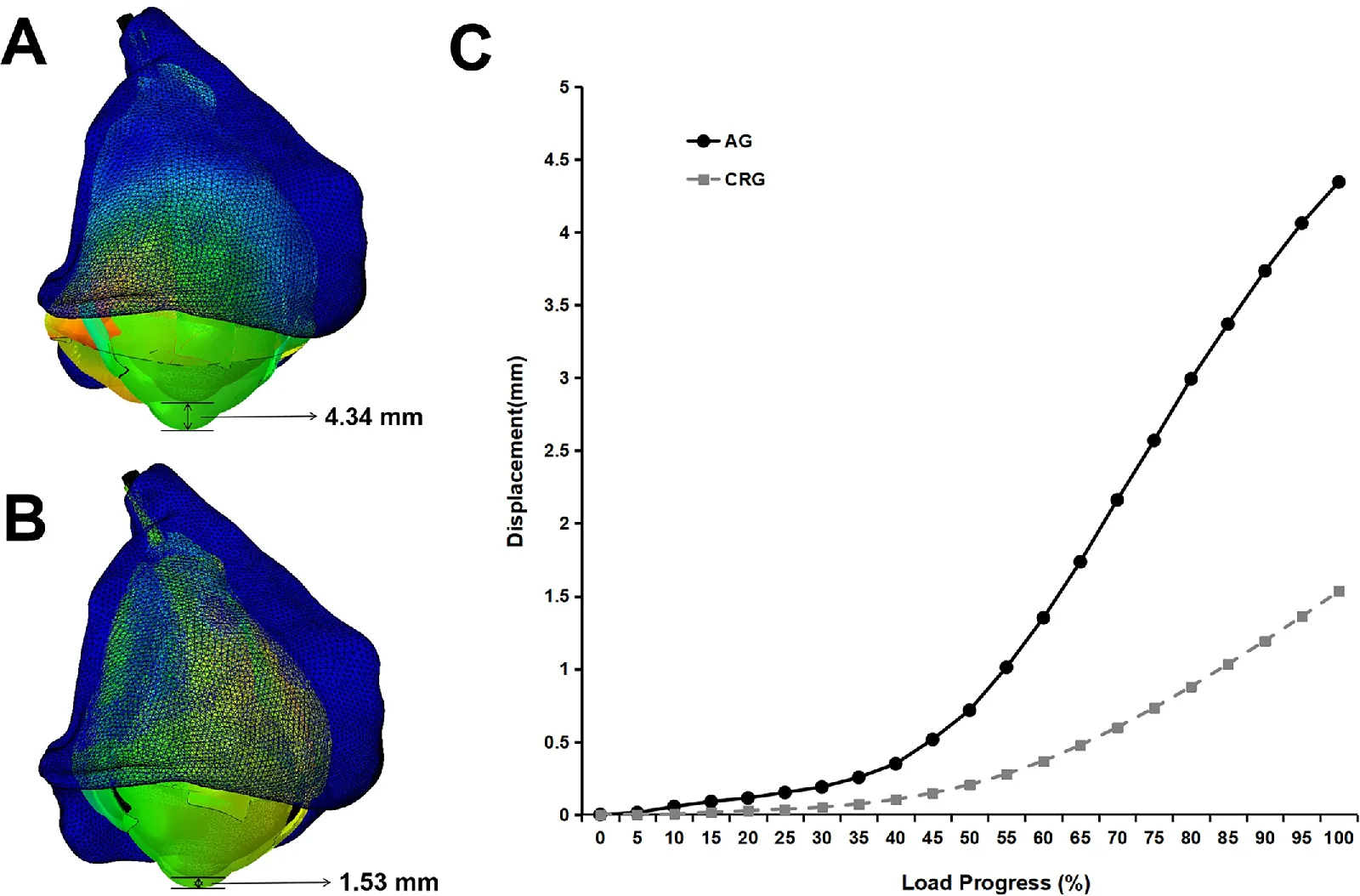
Eye globe displacement analysis for AG and CRG. (**A**) AG globe displacement cloud map (superior-lateral view). The cloud map overlays semi-transparently to compare the initial position of the orbital soft tissues with the final simulated position, highlighting the significant sagittal displacement (proptosis) caused by adipose tissue proliferation. (**B**) CRG globe displacement cloud map (superior-lateral view). The same visualization method is used, showing relatively smaller sagittal displacement caused by muscle hypertrophy. (**C**) Globe displacement curve for the two simulation groups over the entire loading process. The displacement of AG is consistently higher than CRG, and AG shows a nonlinear growth trend, while CRG's growth is relatively smooth.

### Clinical Validation Results

To validate the clinical relevance of the above simulation results, this study retrospectively analyzed the clinical and imaging data of 36 patients with TED. First, following the methodology defined in the Study Participants and Data Collection subsection, 10 healthy control group participants were measured. The mean (SD) EMA/OA ratio for the control group was 0.25 (0.03), and the mean ± two standard deviations was set as the imaging threshold. Based on this imaging reference and combined with clinical functional assessment, the 36 patients with TED were divided into AT (*n* = 18) and MT (*n* = 18). In Figures 6A, 6B, typical CT imaging examples of AT and MT, respectively, show significant hypertrophy of the extraocular muscles in the MT group. Quantitative analysis (Fig. 6C) shows that the mean (SD) EMA/OA ratio for the AT patients was 0.23 (0.04), which did not differ significantly from the control group (*P* > 0.05). The mean (SD) EMA/OA ratio for the MT patients was 0.54 (0.09), significantly higher than both the control and AT groups (*P* < 0.01).

**Figure 6.**
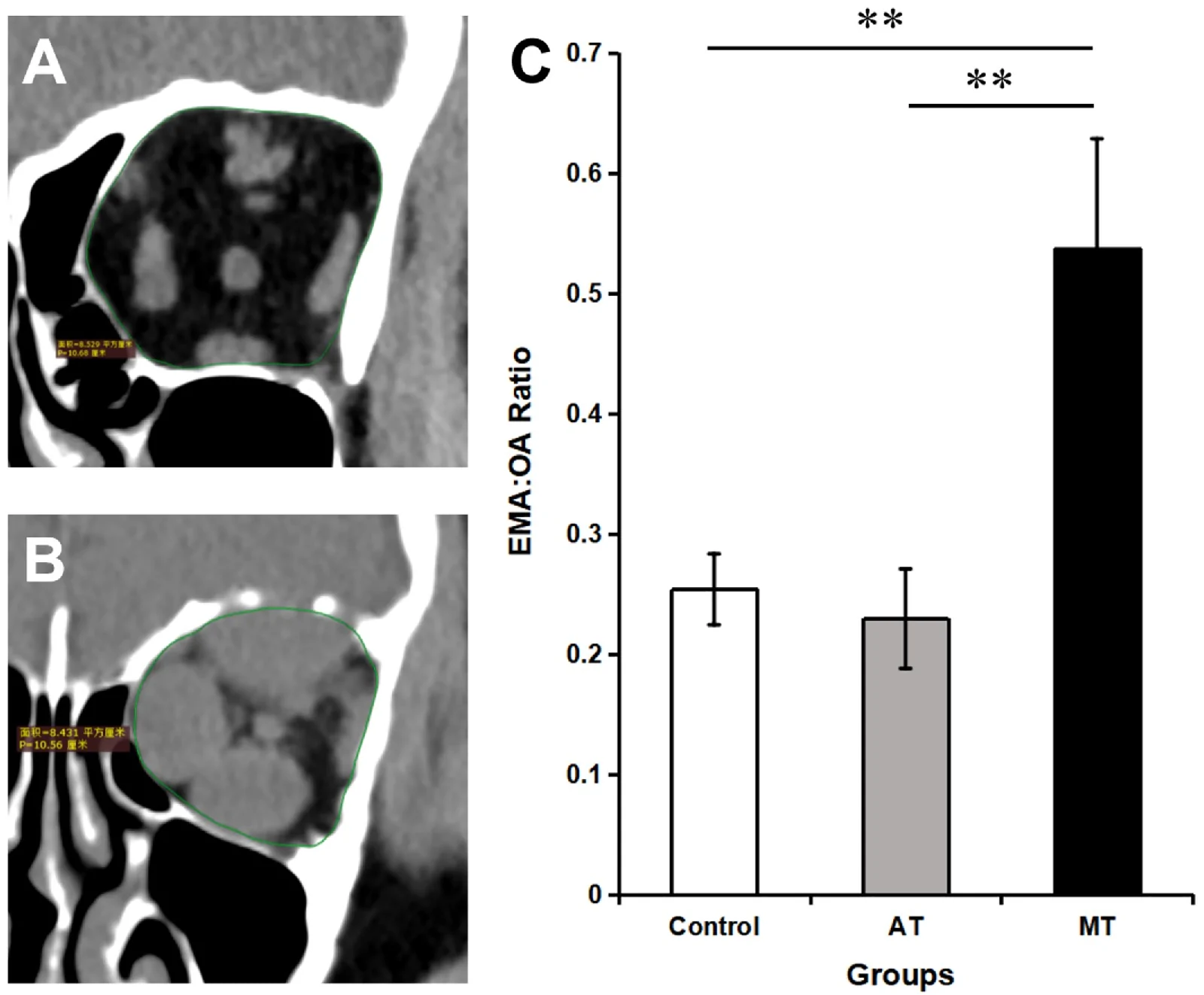
Clinical subtype validation and imaging quantification. (**A**) A typical coronal CT image measurement example from an AT patient, showing medial wall protrusion into the nasal cavity. (**B**) A typical coronal CT image measurement example from an MT patient, showing significant hypertrophy of the extraocular muscles. (**C**) Bar chart of the EMA/OA ratio quantification for the control, AT, and MT groups. The data show that the ratio in the MT group is significantly higher than in the control and AT groups, while there is no statistical difference between the AT and control groups.

In addition, statistical analysis of DON complications in both groups was performed (Table 2). Among the 18 AT patients, 4 (22.2%) had concurrent DON. Among the 18 MT patients, 15 (83.3%) had concurrent DON. A comparison between the groups showed a significant statistical difference in the rate of DON complications (χ^2^ test, *P* < 0.001). This clinical statistic is highly consistent with the simulation results, confirming the effectiveness and clinical relevance of the finite element model in revealing the biomechanical mechanisms underlying TED pathogenesis.

**Table 2.** Correlation Analysis Between TED Clinical Subtypes and DON

Group	With DON	Without DON
Adipose type	4	14
Muscle type	15	3
*P* value	<0.001	

## Discussion

The clinical manifestations of TED exhibit significant diversity, encompassing a range of signs from eyelid retraction and exophthalmos to exposure keratopathy and DON, with the pathologic basis lying in the varying degrees of involvement of the extraocular muscles and intraorbital adipose tissue. Clinically, it is widely believed that significant hypertrophy of the extraocular muscles is closely related to the risk of DON complications.^25^ Imaging studies by Rana et al.^26^ strongly associate muscle enlargement with DON. Nevertheless, the biomechanical mechanisms underlying this association remain incompletely elucidated, particularly the biomechanical effects of simple adipose proliferation leading to DON, and the quantitative differences in optic nerve compression caused by hypertrophy of different rectus muscles.^27^

To explore these issues, this study constructed a 3D orbital model using FEA, aiming to quantitatively compare the pathogenic effects of muscle involvement and simple adipose proliferation. The results revealed two distinctly different biomechanical pathways: hypertrophy of the extraocular muscles leads to high stress on the optic nerve through direct mechanical compression at the orbital apex; conversely, intraorbital adipose proliferation mainly exerts relatively lower stress on the optic nerve through a pulling or displacement mechanism, yet it is the primary factor driving exophthalmos. This dichotomy aligns with mechanistic theories of orbital fat swelling proposed by Jafari et al.^21^ and Jeong et al.^14^ based on orbital FEA models.

The simulation results clearly demonstrate this difference in magnitude: under the same volume expansion ratio, the peak stress on the optic nerve caused by CRG is markedly higher than that caused by AG. The stress cloud map further confirms that the high‑stress area of CRG is concentrated within the muscle cone at the orbital apex and exhibits a ring‑shaped compression pattern. To validate the clinical relevance of this mechanical model, we retrospectively analyzed 36 patients with TED and quantified their pathologic involvement patterns using the EMA/OA ratio method. Clinical statistics found that the patient group primarily affected by muscle involvement indeed had a significantly higher rate of DON complications compared to the group primarily affected by simple adipose proliferation. At the same time, previous studies have also confirmed this through clinical observations.^28^^–^^30^ These clinical observations are highly consistent with the predictions of the simulation model, collectively confirming that significant hypertrophy of the extraocular muscles is a key high‑risk biomechanical factor for the occurrence of DON. Wu et al.^13^ further support that optic nerve stress is higher in muscle‑predominant cases and concentrated at the orbital apex.

In addition to stress analysis, the results of displacement (exophthalmos) analysis presented a contrast: the effect of AG on exophthalmos is far greater than that of CRG. Under the same volume expansion ratio, the sagittal displacement of the eyeball caused by AG is approximately 2.84 times that of CRG. This mechanically explains a common clinical phenomenon: in patients with predominant adipose proliferation, the degree of exophthalmos does not correlate proportionally with the degree of optic nerve compression. The stress cloud map of AG also aligns with this mechanism, as its stress is concentrated at the optic nerve–eye connection rather than the orbital apex, indicating its primary mechanism is the pulling effect caused by adipose proliferation pushing the eyeball forward. Despite this pulling effect, its peak stress is much lower than the apex compression stress caused by muscle involvement. This provides a biomechanical explanation for the phenomenon that many patients with simple exophthalmos (i.e., primarily adipose proliferation) have significant proptosis but a relatively low risk of DON complications. Imaging findings by Kuo et al.^31^ observed that obese patients with TED exhibited significantly greater orbital fat volume and more severe proptosis.

After clarifying the macroscopic differences between muscle and adipose patterns, this study further explored the compressive effects of the four independent rectus muscles: MRG, SRG, LRG, and IRG. Under the same expansion ratio, the peak stress generated by each rectus muscle exhibited a gradient: MRG highest, followed by SRG and LRG, while IRG was the lowest. This stress gradient reveals that different muscles contribute differently to the risk of DON and provides a mechanical explanation for previous clinical observations. For example, Weis et al.^32^ pointed out through volumetric analysis that hypertrophy of the medial rectus muscle is the most relevant independent predictor of DON. Our finite‑element model reveals its intrinsic mechanism from a biomechanical perspective for the first time: the medial rectus muscle, due to its anatomic position closest to the optic nerve at the orbital apex and located on the inner side of the narrow muscle cone, can more effectively transmit mechanical stress to the optic nerve.

The quantitative results of this study have certain guiding significance for clinical decision‑making in TED. The biomechanical foundation provided by this model helps clinicians move beyond traditional qualitative imaging assessments to distinguish between the two pathogenic patterns of compression dominance and exophthalmos dominance. These findings suggest that when formulating individualized surgical plans, appropriate decompression strategies should be selected based on the patient's imaging phenotype. For patients primarily affected by hypertrophy of the extraocular muscles with concurrent DON, since the pathologic core lies in orbital apex compression, treatment should prioritize decompression surgery that fully relieves bony constraints at the orbital apex (especially the medial wall), such as endoscopic medial wall decompression or balanced decompression.^33^^,^^34^ Particularly for those patients with muscle involvement associated with a high risk of DON, the goal of decompression surgery is not only to expand orbital volume but also to completely relieve bony compression at the orbital apex.^35^ This viewpoint also provides biomechanical support for the augmented or functional decompression targeting the orbital apex advocated in recent FEA clinical studies. In contrast, for patients primarily affected by adipose proliferation, presenting as simple exophthalmos, the pathologic core lies in the overall volume increase of the intraorbital contents. In these cases, using a less invasive conjunctival approach for orbital adipose decompression or balanced decompression may be more efficient and appropriate.^36^

This study also has several limitations. First, the model was reconstructed from imaging data obtained from a single healthy young volunteer. Although this standardized model allows the general biomechanical effects of muscle and adipose enlargement to be compared under controlled conditions, it does not account for individual anatomic variations or disease-related tissue and bone remodeling. Therefore, the results should be interpreted as comparative biomechanical evidence rather than direct patient-specific stress predictions or definitive conclusions applicable to all clinical populations.^37^ Second, while EOMs and adipose were modeled with hyperelasticity, the model simplifies the intraocular structures and the multilayered anatomy of the optic nerve (nerve parenchyma vs. sheath) as homogeneous, linearly elastic units. Such simplifications may affect the precision of posterior globe translation and local stress gradients; however, they provide a necessary trade-off for computational feasibility while maintaining our focus on macroscopic pressure transmission at the orbital apex.^38^ In reality, pathologic TED tissues undergo prolonged inflammatory infiltration and fibrosis, resulting in complex constitutive relationships exhibiting nonlinearity, viscoelasticity, and anisotropy; these changes directly affect the transmission and dissipation of mechanical forces.^39^^,^^40^ Furthermore, the model omits the oblique muscles and the intraorbital ligament system, which serve as mechanical transmission and constraint systems within the orbit. Their roles under hypertrophied tissue compression are simplified, potentially affecting the simulation results of stress transmission and deformation within the orbit. Previous quantitative magnetic resonance imaging (MRI) studies^41^ have shown that extraocular muscle volume, fat fraction, and T2-related tissue characteristics can provide additional information on structural changes in the extraocular muscles. These MRI-derived tissue features were not incorporated into the present CT-based mechanical model. In addition, although the present study included four single-rectus hypertrophy groups and a combined rectus hypertrophy group, it did not simulate all clinically observed combinations of muscle involvement, such as selective combined enlargement of the inferior and medial rectus muscles.

Future studies should develop patient-specific finite element models incorporating CT- and MRI-derived geometry and tissue characteristics. Recent developments in multimodality imaging support the integration of complementary structural, functional, and molecular information for more comprehensive tissue characterization.^42^ In parallel, artificial intelligence–assisted analysis of multimodal imaging data may improve automated segmentation, feature extraction, and personalized disease assessment.^43^ Although these approaches have not yet been validated for TED-specific biomechanical modeling, their future integration may facilitate automated orbital reconstruction, disease-specific parameter assignment, and individualized finite element simulation.

## Conclusions

This study employs finite element simulation and clinical validation to quantitatively assess the biomechanical mechanisms of TED. It identifies two distinct biomechanical pathways: hypertrophy of the extraocular muscles results in significant stress on the optic nerve through direct compression at the orbital apex, increasing the risk of DON, whereas intraorbital adipose proliferation primarily drives exophthalmos, exerting relatively lower stress through traction forces. The study also quantifies the compressive effects of individual rectus muscles, with the medial rectus muscle generating the highest mechanical stress, supporting its role as a key contributor to DON risk.

By offering a biomechanical basis for clinical decision-making, this study highlights the importance of distinguishing between compression-dominant and exophthalmos-dominant TED patterns when determining individualized surgical strategies. Future refinement of the model, incorporating patient-specific data and nonlinear material properties, promises to enhance individualized risk assessment and further clarify the biomechanical underpinnings of TED.
